# Daytime Sleepiness and Parkinson's Disease: The Contribution of the Multiple Sleep Latency Test

**DOI:** 10.1155/2014/767181

**Published:** 2014-07-10

**Authors:** Marcelo Ataide, Clélia Maria Ribeiro Franco, Otavio Gomes Lins

**Affiliations:** ^1^Pós-Graduação em Neuropsiquiatria e Ciências do Comportamento, Universidade Federal de Pernambuco, Recife, PE, Brazil; ^2^Hospital das Clinicas, Universidade Federal de Pernambuco, Recife, PE, Brazil

## Abstract

*Background*. Sleep disorders are major nonmotor manifestations of patients with Parkinson's disease (PD), and excessive daytime sleepiness (EDS) is one of the most common symptoms. *Objective*. We reviewed a current literature concerning major factors that influence EDS in PD patients, using Multiple Sleep Latency Test (MSLT). *Methods*. A Medline search found 23 studies. *Results*. The presence of EDS was observed in 12.7% to 47% in patients without complaints of daytime sleepiness and 47% to 66.7% with complaints of daytime sleepiness. Despite being recognized by several authors, major factors that influence EDS, such as severity of motor symptoms, use of dopaminergic medications, and associated sleep disturbances, presented contradictory data. *Conclusions*. Available data suggest that the variability of the results may be related to the fact that it was conducted with a small sample size, not counting the neuropathological heterogeneity of the disease. Thus, before carrying out longitudinal studies with significant samples, careful analysis should be done by assigning a specific agent on the responsibility of EDS in PD patients.

## 1. Introduction

Parkinson's disease (PD) is a leading progressive neurodegenerative disease, with prevalence estimated 1-2% of the population above 55 years. Sleep-related disturbances are frequent in this population and, in some cases, may be the initial manifestation of the disease. Around 60 to 90% of PD patients affected by sleep disorders suffer negative impact on their quality of life [1–3]. A population study, which evaluated 245 PD patients, showed that more than two-thirds of them had sleep-related disturbances and similar complaints were observed in 46% of diabetic patients and 33% of control patients [4]. Then, assessment of sleep is critical for a holistic management strategy of PD. In a Brazilian study, Braga-Neto et al. found that 53.3% of PD patients reported insomnia, 49.9% restless legs syndrome, 55.4% vivid dreams, 71,8% snoring, and 31.5% daytime sleepiness [5].

Excessive daytime sleepiness (EDS) has been recognized as a common symptom in PD patients. It is defined as a difficulty in maintaining alertness in everyday situations, such as watching television, reading or driving, and interfering with the quality of life, professional and social [6]. Studies in the general population showed rates 0.3–16.3% [7]. Depending on the method of evaluation, in patients with PD, prevalence from 15.5 to 74% was found [1].

Daytime sleepiness can be investigated through subjective and objective studies. Subjective evaluations use standardized questionnaires that must be completed by patients, which assess the patients' own perception of their sleepiness. Several sleepiness scales have been published, but the most used is the Epworth Sleepiness Scale (ESS) [8] that has recently been recommended by the Movement Disorders Society [9]. But some problems were identified in this type of subjective assessment, such the ambiguity of the terms used [10] and influenced by psychological factors such as anxiety, depression, and somatization [9].

The Multiple Sleep Latency Test (MSLT) is the technique most widely used, being considered by the American Academy of Sleep Medicine the gold-standard for objective assessment of sleepiness [11]. The interpretation of results is independent of external factors such as self-perception of sleep. This test involves a series of naps in a soporific environment, with an interval of two hours between them. Adult healthy control subjects have a mean sleep latency of 10 to 20 minutes. Sleep latencies between 5 and 10 minutes indicate moderate sleepiness with less well-defined pathology and consequences. But a sleep latency of less than five minutes has been related to impairments in performance of daily tasks, being considered a pathological level of daytime sleepiness. A meta-analysis determined that daytime sleep latency (DSL) in the healthy population is 10.5 ± 4.6 minutes. In patients with pathological sleepiness, such as narcolepsy and idiopathic hypersomnia, sleep latency was 3.1 ± 2.9 and 6.2 ± 3.0 minutes, respectively [12].

The MLST also assesses the sleep-onset REM periods (SOREMPs) during naps. It is defined as one or more epochs of REM sleep occurring within 15 minutes of the first epoch scored as sleep and the presence of two or more SOREMPs is highly specific for the diagnosis of hypersomnias, typically narcolepsy [13]. Studies found a prevalence of two or more SOREMPs varying 3.9% [14] in the general population to 13.1% in men and 5.6% in women [15].

Several authors agree that DSL in PD patients may be influenced by three factors. EDS may be primary to the disease itself, due to loss of both dopaminergic neurons and nondopaminergic neurons involved in the control of the sleep-wake cycle, secondary to nocturnal sleep deprivation from coexistent sleep disorders or to the use of daytime medications with sedative effect, in particular dopaminergic agonists (DA). However, there are contradictory results in the literature.

The aim of this review is to determine the prevalence of SED through the MSLT studies in PD patients as well as possible factors influencing daytime sleep latency. For the purpose of this review we focused on longitudinal studies, but we also summarize the results of cross-sectional studies.

## 2. Materials and Methods

A Medline literature search was performed to identify all studies on sleep disorders and PD published from January 1, 1990, through December 31, 2013, in English, using the MeSH terms: “Multiple Sleep Latency Test,” “MSLT,” “Parkinson's Disease,” and “parkinsonism.” Published abstracts were included, and additional references were taken from article citations.

Following data extraction, we selected 23 studies, longitudinal and cross-sectional (14 in patients without complaints of daytime sleepiness [16–29] and nine in patients with complaints of daytime sleepiness [30–38]) (Tables 1 and 2).

## 3. Results

Studies presented were conducted by tertiary sleep centers or outpatient movement disorders units, both from Europe, Asia, and US. The number of included PD patients was relatively small in all the studies (ranging from 3 to 80) and mean age at examination was 50 or older. The diagnosis of sleep disorders was based on polysomnography or MSLT.

The mean DSL in PD patients widely ranged from 7.2 ± 5.6 [20] to 18 ± 4 [29] minutes in patients without complaints of daytime sleepiness and 4.0 ± 2.5 [35] to 10 ± 5.5 [37] minutes in patients with complaints of daytime sleepiness. In the first group, mean DSL of less than five minutes was observed in 12.7% [27] to 47% [20], and in the second group it was observed in 42% [32] to 66.7% [35].

### 3.1. Case-Control Studies of Mean DSL among PD Patients and Healthy Controls

Quite a few case-control studies have compared mean DSL among PD patients and healthy controls. Three case-control studies [16, 26, 27] compared mean DSL in PD patients without complaints of daytime sleepiness and in healthy patients. In the study by Rye et al., the first study to assess sleepiness in idiopathic PD employing a standard methodology, no differences were seen between the latencies of PD patients and controls (11 ± 6.1 versus 11.8 ± 5.3 minutes, resp., *P* = ns) [16]; the same was observed by Bušková et al. in newly diagnosed untreated PD patients (11.7 ± 4 versus 12.5 ± 2 minutes, resp., *P* = ns) [26].

However, Yong et al. conducted one study involving overnight PSG followed by MSLT on unselected Asian PD patients and observed that the mean DSL was shorter in the control group (9.5 ± 4.2 minutes) than in PD patients (12.5 ± 5.6 min) (*P* = 0.010). EDS was observed in 16.7% of controls and 12.7% of PD patients, with no significant difference between them (*P* = 0.544), despite the fact that PD patients experience more severe daytime sleepiness subjectively as measured by the ESS than controls [27].

### 3.2. Onset of Studies among PD Patients with or without SOREMPs

#### 3.2.1. Cross-Sectional Studies

Six studies reported the presence of SOREMPs during daytime naps in patients with and without previous complaints of daytime sleepiness [18, 30, 32, 33, 35, 37]. The estimated prevalence of two or more SOREMPs in PD patients with pathologic DSL widely ranged (from 10% [30] to 100% [35]). These patients formed the narcolepsy-like group.

Arnulf et al., who investigated the relationship between hallucinations and sleep disorders and daytime and nighttime polysomnographic recordings, observed that patients with hallucinations had lower DSL compared to the group of patients without hallucinations and the presence of SOREMPs was higher in the former compared to the latter [30]. In a new study, the authors, who studied sleep and daytime sleepiness in 54 patients with PD presenting with sleep problems, showed that patients in the narcolepsy-like group had lower DSL than patients in the nonnarcolepsy-like group (4.6 ± 0.9 versus 7.4 ± 0.7 minutes, *P* = 0.02). There were no differences between narcolepsy and nonnarcolepsy-like groups for age, duration of disease, ESS, Unified PD Rating Scale Motor Disability (UPDRS) score, nighttime sleep, daily dose of levodopa, and dose of dopamine agonists. This pattern may be secondary to rapid eye movement (REM) sleep abnormalities like the presence of REM during stage 2 sleep in some patients in the narcolepsy-like group, a pattern also described in primary narcolepsy [32]. In one study to determine if patients taking DA, and reporting undesired unintended sleep episodes, were indeed sleepy during the day, Roth et al. also reported similar results where the mean DSL in patients with SOREMPs was significantly lower than patients without SOREMPs (4.5 ± 2.7 versus 9.1 ± 5.1 minutes, *P* < 0.05) [33].

In four studies [19, 20, 25, 26], no SOREMPs were found on MSLT in PD patients.

#### 3.2.2. Case-Control Studies

Only four studies reported the presence of SOREMPs during daytime naps in patients with and without previous complaints of daytime sleepiness, compared with healthy controls [16, 24, 26] or untreated PD patients [22].

In a case-control study to assess the presence and severity of sleep disturbances in newly diagnosed untreated 20 PD patients, Bušková et al. observed one patient that had a mean DSL in the pathological range (<8 min) and had a single SOREMP on MSLT. Two other patients with normal sleep onset latencies had one SOREMP on MSLT and none of PD patients had multiple SOREMPs. No SOREMP was found on MSLT in controls [26]. A previous study found that patients with SOREMPs had lower mean DSL than patients without SOREMPs (4.2 ± 1.7 versus 13.1 ± 7.0 minutes, *P* < 0.001) and presence of SOREMPs on the MSLT demonstrated hallucinatory symptoms bearing phenotypic similarity to narcolepsy [16].

### 3.3. Onset Studies about Influence of Disease Activity

#### 3.3.1. Cross-Sectional Studies

Three cross-sectional studies conducted on PD patients without previous complaints of daytime sleepiness evaluated the association between the results of score MSLT and parkinsonian motor symptoms [20, 25, 28], stratifying patients by disease duration, as measured by the number of years since diagnosis, or through scales of symptom severity, like Hoehn and Yahr Staging (H&Y) and UPDRS.

None of the studies above showed correlation between DSL versus disease duration or the severity of parkinsonian symptoms. In one study that measured sleepiness and alertness, both subjectively and objectively, in consecutive PD patients not selected for sleepiness or medication use, the duration of disease, the UPDRS, and H&Y did not correlate significantly with the score of MSLT [20], similar results obtained by Chung et al. [28]. Poryazova et al., who assessed characteristics and determinants of EDS in consecutive 30 PD patients, also showed no significant differences regarding disease duration and severity (as expressed by UPDRS and H&Y stage) between patients with mean DSL < 5 minutes compared to patients with mean DSL > 5 minutes, but patients with symptoms of wearing off (36.7%, *n* = 11), a sign of advanced PD, had the lowest mean DSL (5.9 ± 5.8 versus 11 ± 6.1 minutes, *P* < 0.031) [25].

Only one study described the association between the duration and severity of symptoms with the results of the MSLT in patients with complaints of daytime sleepiness. There was no correlation between mean DSL and disease duration (*r* = 0.08), H&Y (*r* = 0.17), and UPDRS (*r* = 0.01) [32].

#### 3.3.2. Case-Control Studies

Two case-control studies [16, 27] also evaluated the correlations between DSL versus disease duration or the severity of parkinsonian symptoms. Data obtained from PD patients indicated that DSL does not undergo influence of these variables. Rye et al. showed no correlation between the presence of daytime sleepiness with the following variables: disease duration and H&Y; however, patients who had one or more SOREMPs had longer disease duration than patients who did not have SOREMPs (11.8 ± 8.4 versus 6.1 ± 4.9 years; *P* < 0.05) [16]. In Asian patients, Yong et al. also observed that mean DSL was not significantly associated with disease duration, H&Y stage, and UPDRS for PD patients [27].

### 3.4. Onset Studies about Influence of Dopaminergic Medications

#### 3.4.1. Cross-Sectional Studies

Two studies [19, 25] conducted on PD patients without complaints of daytime sleepiness evaluated the association of score MSLT with the use of dopaminergic medications, where dosage was a risk factor associated with pathologic DSL.

The study conducted by Razmy et al. that used standard polysomnographic techniques to evaluate daytime sleepiness and wakefulness in 80 PD patients receiving dopamine agonist therapy evaluated the relationship of episodes of drowsiness with the pharmacodynamic differences between the different DA (pramipexole, ropinirole, and bromocriptine/pergolide). The mean DSL did not differ between treatment groups (F2,77 = 0.11; *P* = 0.90). Univariate risk factor (*P* < 0.01) was associated with levodopa dosage equivalents (LDE) (OR, 4.2; 95% CI, 1.3–13.7) and high LDE were found to be independently associated with pathologic DSL [19]. A Swiss study showed that DSL was significantly correlated with the dose of dopamine agonists equivalents (*r* = −0.484, *P* = 0.007); the same was not observed when the LDE (*r* = −0.348, *P* = 0.059) was analyzed, finding a dose-dependent effect with DA [25].

Only one French study described the correlation between the use of dopaminergic medications and complaints of daytime sleepiness. This study observed a weak correlation between DSL and daily dose of levodopa (*r* = 0.3; *P* = 0.03). The mean DSL in patients treated with levodopa alone was similar to those patients treated with levodopa and DA (6.8 ± 0.9 versus 5.8 ± 0.8 min, *P* = 0.44). There was no correlation between score MLST and daily dose of DA (bromocriptine equivalent, *r* = −0.17, *P* = 0.2) or LDE (*r* = 0.17, *P* = 0.2). However, there was a weak positive correlation between means DSL and daily dose of levodopa (*r* = 0.3, *P* = 0.03) [32].

#### 3.4.2. Case-Control Studies

Moreover, three studies [16, 23, 27] found no correlation between daytime sleepiness and dopaminergic dose. Rye et al. observed no significant difference in the presence of daytime sleepiness among patients free of treatment in relation to other treated patients. Comparisons between patients receiving versus those not receiving specific medications indicated that MSLT scores were unassociated with levodopa/carbidopa and amantadine. Selegiline use, on the other hand, was associated with greater sleepiness (5.8 ± 2.5 versus 11.9 ± 6.2 minutes, *P* < 0.07) [16]. When the patients were divided into groups according to levodopa alone or in combination with DA, Shpirer et al. also showed, in 46 PD patients, no differences in daytime sleepiness, measured by MSLT, admitting that several factors play a possible role in the pathogenesis of EDS [23].

#### 3.4.3. Longitudinal Studies

Regarding the daytime sleepiness in 15 PD patients newly diagnosed and had never received antiparkinsonian medication and had not taken other psychotropic, antidepressant drugs or benzodiazepines, Kaynak et al. found that DSL was significantly lower after treatment than obtained before (8.1 ± 4.7 versus 13.6 ± 4 minutes, *P* < 0.005), where a pathologic DSL was reported by only one patient before but was obtained in seven patients (46.6%) after treatment. Of the patients with objective daytime sleepiness, three (42.8%) had two or more SOREMPs periods during MSLT as similar to that seen in narcolepsy-cataplexy syndrome. Two of these patients were on a DA monotherapy and one of them was on dopamine agonist in combination with levodopa. Multiple regression analysis showed that the variable which attained the best explanation for the MSLT score variability was the levodopa dose (Beta-0.01 *P* < 0.05), whereas other variables such use of DA did not show any significance [22].

### 3.5. Onset Studies about Influence of Sleep Disturbances

#### 3.5.1. Cross-Sectional Studies

Five clinical based studies with cross-sectional studies [20, 25, 28, 32, 37] conducted on PD patients examined the occurrence and clinical correlates of sleep-related breathing disorders with score MSLT. The apnea-hypopnea index (AHI) ranged from 8.6 ± 16.5 [20] to 21.7 ± 20.6 [28]/hour. The presence of obstructive sleep apnea syndrome (OSAS) ranged from 33 [25] to 74% [28] of patients.

Although not statistically significant, in an American study, there was a trend for the sleepier subjects to have more severe obstructive sleep apnea with a greater AHI (*r* = −0.34, *P* = 0.15) [20]. Stevens et al. found that patients with DSL less than five minutes had significantly higher AHI (24 ± 9 versus 26 ± 15/hour, *P* = 0.026). Sleepiness on the MSLT was best predicted by sleep fragmentation, although this association is not robust. Although not statistically significant, there was a trend for the sleepier subjects to have more severe obstructive sleep apnea with a greater AHI [25]. In recent study, Chung el al. examined associations of both motor and nonmotor PD symptoms with reported sleep problems. The authors showed that the AHI correlated with objective daytime sleepiness, as reflected by shorter mean sleep latency on the MSLT (rho = −0.47, *P* = 0.003), although the AHI was not significantly correlated with rating scale scores or demographic variables [28].

However, in PD patients with complaints of daytime sleepiness, Arnulf et al., in the first time that the prevalence of sleep-disordered breathing has been measured in sleepy PD patients and where 20% of the patients in this study had a moderate to severe OSAS, found no correlation between the score MSLT with AHI and also observed no differences between patients in the narcolepsy-like group and nonnarcolepsy-like group in relation to intensity of respiratory symptoms (12 ± 16 versus 10 ± 13/h, *P* = 0.6) [32]. Likewise, Monaca et al. showed no correlation between DSL and AHI, although patients had an AHI ≥ 10/h [37].

In relation to other sleep disorders, a recent study showed that the REM sleep behavior disorder (RBD) patients, characterized by the loss of normal REM sleep muscle atonia and the presence of behaviors related to dream content, had a significantly larger mean DSL than the non-RBD patients (18 versus 15 min, resp., *P* = 0.014) [29].

#### 3.5.2. Case-Control Studies

Five different case-control studies' design [16, 22, 24, 26, 27] conducted on PD patients evaluated the association of score MSLT with sleep related-breathing disorders. The AHI ranged from 3,6 ± 4 [22] to 18,0 ± 17,8 [24]/hour. The presence OSAS ranged from 20 [26] to 49.1% [27] of patients. In control patients, these results ranged from 20 [26] to 65.7% [27]. No correlations were found between scores of MSLT and AHI. Despite the fact that 26.9% of PD patients (*n* = 56) with OSAS had abnormal daytime sleepiness, Yong et al. showed that the presence of OSA (and AHI) did not affect the daytime sleepiness experienced by PD patients [27].

## 4. Discussion

The sleep-related clinical features of PD were described by James Parkinson in 1817 in* Essay on the Shaking Palsy*. However, the first polysomnographic recordings of parkinsonian patients were performed in the 1950s and 1960s. Although the pathophysiology of PD involves regulatory control centers of the sleep-wake cycle, no differences were observed between the results of the MSLT in patients with PD and control population, except in the Asian population, where Yong et al. found that control patients were more sleepy [27]. This bias may be associated with reduced size of the sample.

Regarding the presence of SOREMPs during daytime naps, several authors have cited a probable narcolepsy-like syndrome associated with DP, especially when associated with hallucinations. Daytime REM intrusions can cause altered dream perception and be misdiagnosed as hallucinations. It is believed that the massive loss of hypocretinergic cells in the hypothalamus and other areas such as the subcoeruleus induces excessive pressure of REM sleep during the day and contributes to the symptoms of narcolepsy in patients with PD. This loss may be 23% in the early stages, reaching 62% in the later stages of the disease [39]. However, Baumann et al. showed reduced levels of hypocretin-1 in only 20% of study patients without association with daytime sleep latency [35]. Compta et al. did not observe differences between the levels of hypocretin of PD patients and controls and there was no correlation between daytime sleep latency and levels of hypocretin [24].

Beside the reduction of hypocretinergic neurons, other potential mechanisms related to sleep disorders are dysfunction in dopaminergic signaling and accumulation of *α*-synuclein in regions of the brainstem. It is possible that the disruption of dopaminergic signaling causes a failure in the control of wakefulness, resulting in an excessive increase in daytime sleepiness [40]. In the second case, these changes are related to the accumulation of *α*-synuclein and Lewy bodies, with consequent neuronal loss in selective areas of the brain stem involved in the regulation of sleep and wakefulness [40, 41].

In studies with MSLT, regardless of the initial complaint of sleep disturbance, daytime sleep latency showed no correlation with the duration and the intensity of parkinsonian motor symptoms. One of the questions may be the fact that patients are assessed during “on” time and may not correlate with the neuropathological changes. Methodological differences (retrospective versus prospective recruitment and sources of controls) as well as differences in patient characteristics and medications also may partly explain variances in published studies.

The effects of dopaminergic medications can be evaluated in two ways: by the total dose of dopaminergic or the type of medication. The effects of dopaminergic drugs on sleep may be related to dose, showing a biphasic pattern, where lower doses are promoters of slow-wave and REM sleep through D2 autoreceptors, leading to impaired wakefulness and higher doses have the opposite effect, possibly due to differential activation of D1 receptors [2].

Kaynak et al. suggested that sleepiness cannot be attributed to disease progression and the MSLT scores were best explained by high doses of levodopa, suggesting predictive effect of EDS [22]. Razmy et al. observed that only high doses of dopamine agonists were independently associated with EDS [19]. However, the same was not observed in studies in Spanish [24] and Asian populations [27].

Through subjective measure of daytime sleepiness, Gjerstad et al. found that the use of DA, but not the daily levodopa dose, was significantly associated with EDS. The frequency of EDS was nearly as high among patients who had never used dopamine agonists compared to those who used agonists and with the same dramatic increase over the 8-year period [42]. Among studies with MSLT in PD patients, only one observed specific association with the dose of DA [25]. There was no relation to the specific type of medication. This could be attributed to a dependent sedative effect of presynaptic autoreceptors. However, this hypothesis does not seem compatible with the clinical findings where the dosages of DA are relatively high and preferentially stimulate the postsynaptic receptors and the decrease of dose was related to reduction of drowsiness [41]. The lack of association observed in most studies may likely be the result of factors not identified as the regional expression of these receptors, the pre- or postsynaptic location, and affinity of the messengers coupled to receptor subtypes.

The sleep-related breathing disorders are the second most common cause of EDS [43] and the prevalence found in studies with MSLT is in agreement with that of the literature, but the correlations were also contradictory. The influence of OSAS on the EDS was observed in only one study with MSLT [25]. Arnulf et al. found no correlation between the results of the MSLT with AHI as well as no differences between the groups with “narcolepsy-like” symptoms and without “narcolepsy-like” symptoms and the intensity of respiratory symptoms [32]. Similar results were observed in Czech [26] and Chinese [27] patients.

It is believed that the motor phenomena, such as rigidity, diaphragmatic dyskinesia, and autonomic disorders of respiratory control mechanisms can lead to reduced respiratory muscle function causing a restrictive pattern in respiratory dynamics and precipitating sleep apnea. However, these contradictions may be due to the fact that sleep apnea does not represent the main cause of the EDS, due to difficulties in assessing these disorders using conventional methods currently used.

Besides these factors, others may contribute to daytime somnolence in PD patients. Nocturnal insomnia, parasomnias, sedative drugs, and dementia have been suggested to cause EDS.

## Figures and Tables

**Table 1:** Characteristics and results of MSLT studies in patients with Parkinson's disease without complaints of sleepiness (*n*: 14).

Authors (country)	Study design	Diagnostic procedures and rating scales	Results
Rye et al., 2000 (USA) [16]	Population-based (case-control)/*n*: 27	MSLT	There was not smaller daytime sleep latency (DSL) in PD patients compared to controls (11 ± 6.1 versus 11.8 ± 5.3 min). No correlation was observed during daytime sleepiness with the following variables: age, gender, disease duration, and H&Y staging. Six had one or more SOREMPs during the MSLT, being significantly sleepier than others (12.4 ± 6.3 versus 6.0 ± 1.3 min, resp., *P* < 0.0005). The presence of SOREMPs was higher in patients who used selegiline (3/4 versus 3/23, chi-square: 7.5 *P* < 0.001).

Bliwise et al., 2002 (USA) [17]	Population-based (cross-sectional)/*n*: 32 (PD primary: 27; Secondary: 5)	MSLT	The daytime sleep latency was 11.5 ± 6.0 min, with no difference between patients with primary and secondary DP.

Nomura et al., 2003 (Japan) [18]	Population-based (cross-sectional)/(*n*: 22) MSLT: 3	MSLT	The MSLT was performed in patients with hallucinations. All feature daytime sleep latencies that exceeded 10 minutes (12.9 ± 2.1 min), however the presence of two or more SOREMPs. The REM latency ranged from 0.9 to 6.1 minutes.

Razmy et al., 2004 (Canada) [19]	Population-based (cross-sectional)/*n*: 80 (pramipexole: 29, ropinirole: 28 e bromocriptine/pergolide: 23)	MSLT, MWT	The mean DSL was 12.1 ± 5.1 min, where 15 (18.8%) exhibited excessive daytime sleepiness (MSLT < 5 min). Presence of an association of univariate risk factor (*P* < 0.10) between the pathological daytime sleepiness and levodopa dosage equivalents (OR, 4, 2; 95% CI. 1,3–13,7).

Stevens et al., 2004 (USA) [20]	Population-based (cross-sectional)/*n*: 20	PSG, MSLT, MWT	The mean DSL was 7.2 ± 5.6 minutes and nine patients (47%) had latency less than 5 minutes. Around 74% of the sample had mean score of MSLT below 10 minutes. The strongest predictor of score MSLT was percentage of stage 1 sleep, suggesting that decreased quality of sleep plays a role in increased sleepiness in PD patients. Specifically, an increased pergolide equivalent was a very strong predictor of increased sleepiness, with a correlation of −0.70 (*P* < 0.0008).

Möller et al., 2005 (Germany) [21]	Population-based (cross-sectional)/*n*: 20	MSLT	There were no differences in DSL between patients with or without sleep attacks (11.6 ± 5.6 versus 14.6 ± 4.9, resp., *P* = 0.22).

Kaynak et al., 2005 (Turkey) [22]	Population-based (cross-sectional)/*n*: 15 (before and after treatment)	TMLS	The mean DSL was significantly lower after treatment than before treatment (8.1 ± 4.7 versus 13.6 ± 4 min, resp., *P* < 0.005) (duration of treatment: 9.8 ± 2 months). Three patients with EDS (42.8%) had two or more SOREMPs after treatment. Multiple regression analysis showed that the best explanation for the variability of the MSLT was the dose of levodopa (beta = 0.01, *P* < 0.05).

Shpirer et al., 2006 (Israel) [23]	Population-based (case-control)/DP: 46 (controls: 30)	MLST	The mean DSL was 14.9 ± 6.9 minutes. No difference was found in daytime sleepiness, measured by MSLT, between patients treated with levodopa alone and patients treated with a combination of levodopa and dopaminergic agonist.

Compta et al., 2009 (Spain) [24]	Population-based (case-control)/*n*: 41 (dementia: 20, without dementia: 21) (Controls: 22) MSLT: 15	MSLT, vPSG, Hypocretin-1	There were no differences in mean DSL between PD patients with and without dementia (in seconds: 481.0 ± 361.9 versus 484.5 ± 387.1; *P* = 0.81). SOREMPs were observed in two PD patients without dementia (one with a normal and one with an abnormal sleep pattern) and two PD patients with dementia (both with abnormal sleep pattern). There was no relation between the AHI and the MSLT latency. The mean DSL did not show any correlation with the CSF hypocretin-1 levels (Spearman test: *r* = 0.49; *P* = 0.10).

Poryazova et al., 2010 (Switzerland) [25]	Population-based (cross-sectional)/*n*: 30	ESE, TMLS, PSG	The mean DSL was 9.2 ± 6.4 minutes. Eleven patients (37%) had severe objective EDS, which is latency <5 minutes. Patients with wearing-off symptoms (*n* = 11) had lower latency (5.98 ± 5.8 versus 11.8 ± 6.1 min, *P* = 0.031). Patients with DSL ≤ 5 minutes had higher AHI (24 ± 26 versus 9 ± 15/h, *P* = 0.026) than patients with DSL > 5 minutes. In 15 patients (50%), a sleep-wake misperception was present. They had significantly longer latency (17.2 ± 3.8 min) in comparison to the rest of the population (7.5 ± 5.6 min, *P* < 0.001).

Bušková et al., 2011 (Czech Republic) [26]	Population-based (case-control)/*n*: 20 (controls: 30) MSLT: 15	MSLT, PSG	The mean DSL did not differ between patients and controls (11.7 ± 4 versus 12.5 ± 2 min, n.s.). In three patients and in none of controls, the mean latency of falling asleep was in the pathological range (8 min). Of these, one had a SOREMP on MSLT. No SOREMP was found on MSLT in controls. No correlations were found between MSLT and AHI.

Yong et al., 2011 (Singapura) [27]	Population-based (case-control)/*n*: 56 (controls: 68)	MSLT, PSG	The mean DSL was lower in controls (9.5 ± 4.2 min) than in PD patients (12.5 ± 5.6 min) (*P* = 0.010). Abnormal daytime sleepiness (DSL < 8 min) was found in 39.4% of controls and 23.6% of PD patients (*P* = 0.208). Around 16.7% of controls and 12.7% of PD patients had severe ESD (DSL < 5 min) (*P* = 0.544). No SOREMP were observed in any of the naps of PD patients. For PD patients, mean sleep latency was not significantly associated with age, gender, disease duration, H&Y stage, UPDRS, LED, or dopamine use.

Chung et al., 2013 (USA) [28]	Population-based (cross-sectional)/*n*: 128 MSLT: 38	ESE, MSLT, PSG	The mean sleep latency in MSLTs was 8.4 ± 5.1 min and it was not significantly correlated with demographic variables of subjects and various rating scales scores. No differences between low and high ESS groups reached significance. However, the AHI correlated with objective daytime sleepiness, as reflected by shorter mean sleep latency on the MSLT (rho = −0.47, *P* = 0.003).

Plomhause et al., 2013 (France) [29]	Population-based (cross-sectional)/*n*: 57	MSLT, PSG	The non-REM sleep behavior disorder (RBD) patients had a significantly shorter mean daytime sleep latency than the RBD patients (15 versus 18 min, resp., *P* = 0.014). None of the MSLTs featured two or more SOREMPs.

DSL: daytime sleep latency; PD: Parkinson's disease; MSLT: Multiple Sleep Latency Test; SOREMP: sleep-onset REM period; EDS: excessive daytime sleepiness; PSG: polysomnography; MWT: Maintenance of Wakefulness Test; H&Y: Hoehn and Yahr; UPDRS Unified Parkinson's Disease Rating Scale; LED: levodopa dosage equivalents; AHI: Apnea Hypopnea Index; RBD: REM sleep behavior disorder.

**Table 2:** Characteristics and results of MSLT studies in patients with Parkinson's disease with complaints of sleepiness (*n*: 9).

Authors (Country)	Study design	Diagnostic procedures and rating scales	Results
Arnulf et al., 2000 (France) [30]	Population-based (cross-sectional)/*n*: 18 (hallucinations: 8/without hallucinations: 10)	MSLT	The mean DSL was shorter in patients with hallucinations compared to the control group (8 ± 1 versus 11 ± 1 min). The presence of SOREMPs was higher in patients with hallucinations (1,9 ± 1,5 versus 0,4 ± 1).

Möller et al., 2002 (Germany) [31]	Population-based (cross-sectional)/*n*: 6 (sudden onset of sleep)	MSLT	The mean DSL was 9.4 ± 3.2 minutes, and four (66.7%) had latency <10 minutes. Daytime sleepiness was primarily a result of the disruption of nocturnal sleep.

Arnulf et al., 2002 (France) [32]	Population-based (cross-sectional)/*n*: 54	MSLT, PSG	The mean DSL was 6.3 ± 0.6 minutes. Latency was less than 5 minutes in 27 patients (50%). Twenty-one patients (39% of the group) met the polysomnographic criterion used to define narcolepsy (i.e., two or more SOREMPs). The daytime sleep latency was shorter in the narcolepsy-like group (4.6 ± 0.9 minutes) than in the nonnarcolepsy-like group (7.4 ± 0.7 minutes, *P* = 0.02). There was no correlation between the DSL and age, disease duration, H&Y staging, UPDRS, and AHI. There was no correlation between the DSL and daily dose of dopamine agonists (*r* = −0.17, *P* = 0.20) or LDE (*r* = 0.17, *P* = 0.20). There was a weak positive correlation between the latency and daily dose of levodopa (*r* = 0.3, *P* = 0.03).

Roth et al., 2003 (USA) [33]	Population-based (cross-sectional)/*n*: 24 (Sudden onset of sleep: 16/without sudden onset of sleep: 8)	MSLT	The mean DSL was 7.2 ± 5.1 minutes in the group with sudden onset of sleep and 8.7 ± 4.8 minutes in the group without sudden onset of sleep (*P* = 0.489). Excessive daytime sleepiness (EDS) was observed in 42% of patients. There was no significant difference between the three types of dopamine agonists (pergolide, pramipexole, and ropinirole) compared to daytime sleep latencies.

Merino-Andreu et al., 2003 (France) [34]	Population-based (cross-sectional)/*n*: 47	MSLT	There was no difference between DSL among patients with accurate perception of naps (28/55%) and patients with false perception of naps (23/45%) (6.0 ± 0.5 versus 7.1 versus 0.7 min, resp., *P* = n.s.).

Baumann et al., 2005 (Italy) [35]	Population-based (cross-sectional)/*n*: 14 (PD primary: 10)	ESE, TMLS, PSG	The mean DSL of PD patients was 4.0 ± 2.5 minutes and seven patients had latency <5 minutes. Only two (14%) patients had SOREMPs, and one met criteria for narcolepsy. There was no association between the levels of hypocretin and daytime sleep latency. The sleep-related breathing disorders contributed to sleepiness in patients with parkinsonism.

Ondo et al., 2005 (USA) [36]	Double blind, placebo controlled trial (Modafenil: 19, placebo: 18)	MSLT	The mean DSL in the group using modafenil was initially 6.4 ± 5.1 minutes and after 4.9 ± 3.6 minutes. In the group receiving placebo, daytime sleep latency was initially 4.5 ± 3.9 minutes and after 4.1 ± 3.4 minutes. MSLT results were not significantly different although the scores worsened less with modafinil (−0.16 ± 3.59 minutes) than with placebo (−0.70 ± 3.28 minutes) (*P* = 0.14).

Monaca et al., 2006 (France) [37]	Population-based (cross-sectional)/*n*: 36	MSLT, PSG	The mean DSL was 10 ± 5.5 minutes. During the nap, 10 parkinsonian patients (3/5 patients with unintended sleep episodes) presented ≥2 SOREMPs. Patients with ≥2 SOREMPs have significantly lower MSLT results compared with patients with ≤1 SOREMPs (5 ± 3 versus 12 ± 5 min, resp.). No correlation was found between MSLT and sleep data.

Dusek et al., 2010 (Czech Republic) [38]	Population-based (cross-sectional)/*n*: 33 MSLT: 8	MSLT, PSG	The mean DSL in ropinirole immediate-release group was 7.4 ± 4 minutes and ropinirole prolonged-release group was 7 ± 6 minutes. There were no differences in the mean sleep latency according to MSLT. No SOREMPs were recorded.

DSL: daytime sleep latency; PD: Parkinson's disease; MSLT: Multiple Sleep Latency Test; SOREMP: sleep-onset REM period; EDS: excessive daytime sleepiness; PSG: polysomnography; H&Y: Hoehn and Yahr; UPDRS: unified Parkinson's disease rating scale; LED: levodopa dosage equivalents; AHI: Apnea Hypopnea Index.

## References

[1] Iranzo A (2006). Parkinon’s disease and sleepiness. *Sleep Medicine Clinics*.

[2] Chaudhuri KR, Schapira AH (2009). Non-motor symptoms of Parkinson's disease: dopaminergic pathophysiology and treatment. *The Lancet Neurology*.

[3] Videnovic A, Comella CL, Aminoff MJ, Boller F, Swaab DF (2011). Sleep disorders in Parkinson’s disease. *Handbook of Clinical Neurology*.

[4] Tandberg E, Larsen JP, Karlsen K (1998). A community-based study of sleep disorders in patients with Parkinson's disease. *Movement Disorders*.

[5] Braga-Neto P, da Silva-Júnior FP, Sueli Monte F, de Bruin PFC, de Bruin VMS (2004). Snoring and excessive daytime sleepiness in Parkinson's disease. *Journal of the Neurological Sciences*.

[6] Arnulf I, Leu-Semenescu S (2009). Sleepiness in Parkinson's disease. *Parkinsonism and Related Disorders*.

[7] Ohayon MM (2006). Epidemiology of excessive daytime sleepiness. *Sleep Medicine Clinics*.

[8] Johns MW (1991). A new method for measuring daytime sleepiness: the Epworth sleepiness scale. *Sleep*.

[9] Högl B, Arnulf I, Comella C (2010). Scales to assess sleep impairment in Parkinson's disease: critique and recommendations. *Movement Disorders*.

[10] Santamaria J (2004). How to evaluate excessive daytime sleepiness in Parkinson's disease. *Neurology*.

[11] Littner MR, Kushida C, Wise M (2005). Practice parameters for clinical use of the multiple sleep latency test and the maintenance of wakefulness test. *Sleep*.

[12] Sullivan SS, Kushida CA (2008). Multiple sleep latency test and maintenance of wakefulness test. *Chest*.

[13] American Academy of Sleep Medicine (2005). *International Classification of Sleep Disorders: Diagnostic and Coding Manual*.

[14] Singh M, Drake CL, Roth T (2006). The prevalence of multiple sleep-onset REM periods in a population-based sample. *Sleep*.

[15] Mignot E, Lin L, Finn L (2006). Correlates of sleep-onset REM periods during the Multiple Sleep Latency Test in community adults. *Brain*.

[16] Rye DB, Bliwise DL, Dihenia B, Gurecki P (2000). Daytime sleepiness in Parkinson's disease. *Journal of Sleep Research*.

[17] Bliwise DL, Rye DB, Dihenia B, Gurecki P (2002). Greater daytime sleepiness in subcortical stroke relative to Parkinson's disease and Alzheimer's disease. *Journal of Geriatric Psychiatry and Neurology*.

[18] Nomura T, Inoue Y, Mitani H, Kawahara R, Miyake M, Nakashima K (2003). Visual hallucinations as REM sleep behavior disorders in patients with Parkinson's disease. *Movement Disorders*.

[19] Razmy A, Lang AE, Shapiro CM (2004). Predictors of impaired daytime sleep and wakefulness in patients with Parkinson disease treated with older (Ergot) versus newer (Nonergot) dopamine agonists. *Archives of Neurology*.

[20] Stevens S, Comella CL, Stepanski EJ (2004). Daytime sleepiness and alertness in patients with Parkinson disease. *Sleep*.

[21] Möller JC, Rethfeldt M, Körner Y (2005). Daytime sleep latency in medication-matched Parkinsonian patients with and without sudden onset of sleep. *Movement Disorders*.

[22] Kaynak D, Kiziltan G, Kaynak H, Benbir G, Uysal O (2005). Sleep and sleepiness in patients with Parkinson's disease before and after dopaminergic treatment. *European Journal of Neurology*.

[23] Shpirer I, Miniovitz A, Klein C (2006). Excessive daytime sleepiness in patients with parkinson's disease: a polysomnography study. *Movement Disorders*.

[24] Compta Y, Santamaria J, Ratti L (2009). Cerebrospinal hypocretin, daytime sleepiness and sleep architecture in Parkinson's disease dementia. *Brain*.

[25] Poryazova R, Benninger D, Waldvogel D, Bassetti CL (2010). Excessive daytime sleepiness in parkinson's disease: characteristics and determinants. *European Neurology*.

[26] Bušková J, Klempíř J, Majerová V (2011). Sleep disturbances in untreated Parkinson's disease. *Journal of Neurology*.

[27] Yong MH, Fook-Chong S, Pavanni R, Lim LL, Tan EK (2011). Case control polysomnographic studies of sleep disorders in Parkinson's disease. *PLoS ONE*.

[28] Chung S, Bohnen NI, Albin RL, Frey KA, Müller ML, Chervin RD (2013). Insomnia and sleepiness in Parkinson disease: associations with symptoms and comorbidities. *Journal of Clinical Sleep Medicine*.

[29] Plomhause L, Dujardin K, Duhamel A (2013). Rapid eye movement sleep behavior disorder in treatment-naïve Parkinson disease patients. *Sleep Medicine*.

[30] Arnulf I, Bonnet A-M, Damier P (2000). Hallucinations, REM sleep, and Parkinson's disease: a medical hypothesis. *Neurology*.

[31] Möller JC, Stiasny K, Hargutt V (2002). Evaluation of sleep and driving performance in six patients with Parkinson's disease reporting sudden onset of sleep under dopaminergic medication: a pilot study. *Movement Disorders*.

[32] Arnulf I, Konofal E, Merino-Andreu M (2002). Parkinson's disease and sleepiness: an integral part of PD. *Neurology*.

[33] Roth T, Rye DB, Borchert LD (2003). Assessment of sleepiness and unintended sleep in Parkinson's disease patients taking dopamine agonists. *Sleep Medicine*.

[34] Merino-Andreu M, Arnulf I, Konofal E, Derenne JP, Agid Y (2003). Unawareness of naps in Parkinson's disease and in disorders with excessive daytime sleepiness. *Neurology*.

[35] Baumann C, Ferini-Strambi L, Waldvogel D, Werth E, Bassetti CL (2005). Parkinsonism with excessive daytime sleepiness: a narcolepsy-like disorder?. *Journal of Neurology*.

[36] Ondo WG, Fayle R, Atassi F, Jankovic J (2005). Modafinil for daytime somnolence in Parkinson's disease: double blind, placebo controlled parallel trial. *Journal of Neurology, Neurosurgery and Psychiatry*.

[37] Monaca C, Duhamel A, Jacquesson JM (2006). Vigilance troubles in Parkinson's disease: a subjective and objective polysomnographic study. *Sleep Medicine*.

[38] Dusek P, Busková J, Růzicka E (2010). Effects of ropinirole prolonged-release on sleep disturbances and daytime sleepiness in Parkinson disease. *Clinical Neuropharmacology*.

[39] Thannickal TC, Lai Y, Siegel JM (2007). Hypocretin (orexin) cell loss in Parkinson's disease. *Brain*.

[40] Rothman SM, Mattson MP (2012). Sleep disturbances in Alzheimer's and Parkinson's diseases. *NeuroMolecular Medicine*.

[41] Gjerstad MD, Alves G, Wentzel-Larsen T, Aarsland D, Larsen JP (2006). Excessive daytime sleepiness in Parkinson disease: is it the drugs or the disease?. *Neurology*.

[42] Arnulf I (2005). Excessive daytime sleepiness in parkinsonism. *Sleep Medicine Reviews*.

[43] Partinen M, Aminoff MJ, Boller F, Swaab DF (2011). Epidemiology of sleep disorders. *Handbook of Clinical Neurology*.

